# Metagenomic Next Generation Sequencing in the Detection of Pathogens in Cerebrospinal Fluid of Patients After Alternative Donor Transplantation: A Feasibility Analysis

**DOI:** 10.3389/fcimb.2021.720132

**Published:** 2021-09-14

**Authors:** Binglei Zhang, Jian Zhou, Ruirui Gui, Zhen Li, Yingling Zu, Juan Wang, Fengkuan Yu, Yanli Zhang, Huifang Zhao, Zhenyu Ji, Yongping Song

**Affiliations:** ^1^School of Basic Medical Sciences, Zhengzhou University, Zhengzhou, China; ^2^Department of Hematology, Affiliated Cancer Hospital of Zhengzhou University and Henan Cancer Hospital, Zhengzhou, China; ^3^Henan Academy of Medical and Pharmaceutical Sciences, Zhengzhou University, Zhengzhou, China; ^4^Academy of Medical Sciences, Zhengzhou University, Zhengzhou, China

**Keywords:** metagenomics next generation sequencing (mNGS), alternative donor, hematopoietic stem cell transplantation, cerebrospinal fluid, pathogen

## Abstract

Central nervous system (CNS) complications can occur in 9%–15% of patients after allogeneic hematopoietic stem cell transplantation (allo-HSCT). The clinical manifestations of the CNS complications are non-specific, with most of them being disturbances of consciousness, convulsions, headaches, fever, and epilepsy, making it difficult to infer the cause of the complications based on clinical manifestations. We retrospectively analyzed the sensitivity and feasibility of metagenomic next generation sequencing (mNGS) in the diagnosis of CNS infections after allo-HSCT. Lumbar punctures were performed on 20 patients with CNS symptoms after receiving alternative donor HSCT(AD-HSCT) at the Affiliated Cancer Hospital of Zhengzhou University from February 2019 to December 2020, and their cerebrospinal fluid (CSF) was collected. The mNGS technique was used to detect pathogens in the CSF. Routine CSF testing, biochemical analyses, G experiments, GM experiments, ink staining, acid-fast staining, and bacterial cultures were carried out, and quantitative PCR (qPCR) tests were used to detect cytomegalovirus (CMV), Epstein-Barr virus (EBV), BK polyomavirus (BKPyV), and human alphaherpesvirus (HHV). A total of 29 tests were performed with 21 of them being positive. Of the five negative patients, three were diagnosed with a posterior reversible encephalopathy syndrome, one as having transplantation-associated thrombotic microangiopathy, and one with transient seizure caused by hypertension. Fifteen patients tested positive, of which four had single infections and eleven had mixed infections. Five cases of fungal infections, six cases of bacterial infections, and 13 cases of viral infections were detected. Among the 13 cases of viral infections, ten cases were CMV(HHV-5); three were BKPyV; two were Torque teno virus (TTV); Two were HHV-1,two were EBV(HHV4), and one each of HpyV5 and HHV-6B. Thirteen patients tested positive for virus while the qPCR detection method of 6 identical specimens were below the minimum detection limit(<1×10^3^ U/ml). The mNGS technique is highly sensitive, and it can be used to diagnose CNS infections after allo-HSCT.

## 1 Introduction

Central nervous system (CNS) complications can occur in 9%–15% of patients after allogeneic hematopoietic stem cell transplantations (allo-HSCT) (Schmidt-Hieber et al., 2016; Das et al., 2020) with the common causes being mainly the posterior reversible encephalopathy syndrome (PRES), transplant-related thrombotic microangiopathies (TA-TMA), CNS graft *versus* host disease (CNS-GVHD), CNS infiltration of malignant disease, CNS infections and non-specific neurological symptoms (Chaudhary et al., 2017; Maffini et al., 2017; Balaguer-Rosello et al., 2019). Furthermore, the clinical manifestations of CNS complications are non-specific, with most of them being disturbances of consciousness, convulsions, headaches, fever, and epilepsy. While it is difficult to infer the cause of the complications based on clinical manifestations. At present, CNS infections are still the main cause of CNS complications after transplantation, with an incidence rate of up to 15% (Schmidt-Hieber et al., 2016). Moreover, the incidence of allo-HSCT is significantly higher than that of autologous HSCT, with the fatality rate also being high (Chaudhary et al., 2017). This is a risk factor for poor prognosis in patients after allo-HSCT (Dowling et al., 2018). For CNS infections after transplantation, traditional detection methods cannot meet clinical needs by offering a quick and accurate means to diagnose the infections and the types of infectious pathogens. Therefore, rapid and accurate detection methods for pathogenic microorganisms have become the focus of current research.

As an emerging detection method, metagenomic next generation sequencing (mNGS) was first recognized for its success in the diagnosis of CNS infectious diseases. The sensitivity of mNGS was more than 90% in untreated patients and 66.67% in patients receiving empirical treatment with a specificity as high as 100%. Since the test results are less affected by antibiotics and other drugs, they can also be used to evaluate the effects of treatment and the monitoring of diseases (Zhang et al., 2019; Zhang et al., 2020). Furthermore, there have been many reports of successful cases that have used the mNGS method in the diagnosis of CNS infections (Wilson et al., 2014; Wilson et al., 2018). Research has shown that the mNGS method also has a high diagnostic value in children with unexplained encephalitis (Haston et al., 2020). In addition, multi-center prospective clinical trials have confirmed its application value in clinical diagnoses (Xing et al., 2020). However, there are few studies on the use of the mNGS method in CNS infections after allo-HSCT. Therefore, we analyzed the sensitivity and feasibility of the mNGS method in the diagnosis of CNS infections after allo-HSCT to provide a reference for the diagnosis and guidance for treatment.

## 2 Methods

### 2.1 General Information

The study protocol was approved by the Ethics Committee of the Affiliated Cancer Hospital of Zhengzhou University, and the study was carried out in accordance with the Declaration of Helsinki. We retrospectively analyzed the data of 20 patients with CNS symptoms who received AD-HSCT at the Affiliated Cancer Hospital of Zhengzhou University between February 2019 and December 2020. The clinical data of the patients were recorded, and the related contraindications excluded. Lumbar punctures were performed under aseptic conditions, and CSF specimens were collected, stored at a low temperature, and tested within six hours. The mNGS method was used to detect pathogens in the CSF. Routine CSF testing, biochemical analyses, G experiments(There is no fungal infection below 70 pg/ml, 70-95 pg/ml is the observation period, continuous testing is given, and deep fungal infection is suspected if it is greater than 95pg/ml), GM experiments(Less than 0.65 ug/L is negative, greater than 0.85 ug/L is positive), ink staining, acid-fast staining, and bacterial cultures were performed, and quantitative PCR (qPCR) was used to detect the cytomegalovirus (CMV), Epstein-Barr virus (EBV), BK polyomavirus (BKPyV), and human alphaherpesvirus (HHV). At the same time, neuroimaging examinations (such as MRI and CT) and other means were used to assist the diagnosis. All the patients provided informed consent for their inclusion in the study. For the children, their parents and guardians provided informed consent.

### 2.2 Methods and Process of mNGS

#### 2.2.1 Sample Processing and Sequencing

2mL CSF was inactived at 80°C for 10 minutes immediately after collection. 1.5mL microcentrifuge tube with 0.8mL sample and 2 g 0.5 mm glass beads were attached to a horizontal plat form on a vortex mixer and agitated vigorously at 3000 RPM for 15 minutes, all Sample was then centrifuged at 12000 RPM for 1 minute, 0.6 mL sample was separated into a new 2.0mL microcentrifuge tube and DNA was extracted using the Humoral microbial DNA Kit (PMD101, Nanjing Practice Medicine Diagnostics Co., Ltd) according to the manufacturer’s recommendation.

According to the protocol of the BGISEQ-200 sequencing platform, the DNA library was constructed through DNA fragmentation, end-repair, adapter-ligation, and PCR amplification. The constructed library was qualified by Agilent 2100 (Agilent Technologies, USA) and Qubit 4.0 (Thermo Fisher, USA). The qualified double-strand DNA library was transformed into a single-stranded circular DNA library through DNA-denaturation and circularization. DNA nanoballs (DNBs) were generated from single-stranded circular DNA using rolling circle amplification (RCA). The DNBs were qualified using Qubit 4.0. Qualified DNBs were loaded into the flow cell and sequenced (50 bp, single-end) on the BGISEQ-200 platform.

#### 2.2.2 Bioinformatic Analysis

High-quality sequencing data were generated by removing low-quality and short (length<35 bp) reads using fastp software (Chen et al., 2018), followed by computational subtraction of human host sequences mapped to the human reference genome (hg38) using STAR alignment (Dobin et al., 2013). After the removal of low complexity and duplicated reads using PRINSEQ algorithms (Schmieder and Edwards, 2011), the remained data were classified by simultaneously aligning to in-house microbial genome databases, consisting of viruses, bacteria, fungi, and parasites, which were mainly downloaded from NCBI (ftp://ftp.ncbi.nlm.nih.gov/genomes/) using Kraken2 software (Wood et al., 2019). The sequencing data list was analyzed in terms of species-specific read number (SSRN), reads per million (RPM) and genome coverage (%).

#### 2.2.3 Threshold Criteria for Interpretation of Metagenomic Analysis

The microbial list obtained from the above analysis process was compared with an in-house background database, which contains microorganisms appearing in more than 50% samples in the laboratory in the past three months. The suspected background microorganisms were removed from the microbial list.

For different types of microbes, the thresholds were set as follows: Extracellular bacteria/Fungus (excluding *Cryptococcus*)/Parasites: SSRN≥30 (RPM≥1.5), ranked among the top 10 for bacteria, fungi, or parasites. Organisms detected in the negative control group or that were present in≥25% of samples from the previous 30 days were excluded but only if the detected SSRN was≥10-fold than that in the negative control group or other organisms. Additionally, organisms present in≥75% of samples from the previous 30 days were excluded. (1) Intracellular Bacteria (excluding *Mycobacterium tuberculosis* and *Brucella*)/Cryptococcus: SSRN≥10 (RPM≥0.5), ranked among the top 10 for bacteria or fungi. Pathogens detected in the negative control group or that were present in≥25% of samples from the previous 30 days were excluded but only if the detected SSRN was≥10-fold than that in the negative control group or other organisms. (2) Virus/*Brucella*: SSRN≥3 (RPM≥0.15), Pathogens detected in the negative control group were excluded but only if the detected SSRN was≥10-fold than that in the negative control group. (3) *Mycobacterium tuberculosis*: SSRN≥1 (RPM≥0.05) (Xing et al., 2020).

## 3 Results

### 3.1 Characteristics of the Patients

Among the 20 patients, ten were male and 10 were female. The median age was 12 years (range, 3 – 56 years), seven had severe aplastic anemia (SAA), seven acute lymphoblastic leukemia (ALL), and four acute myeloid leukemia (AML), including one case of chronic myeloid leukemia (CML) with acute myeloid degeneration and two were myelodysplastic syndrome (MDS). Ten cases accepted unrelated donor transplantation, and ten haploidentical transplantation. There were 12 cases with positive genes mutation/fusion genes and eight with complex chromosomal karyotypes. All the patients underwent peripheral blood stem cell transplantation and high-resolution HLA testing. Three cases were HLA10/10 compatible, six were HLA9/10 compatible, four were HLA8/10 compatible, one was HLA6/10 compatible, and the HLA5/10 matched six cases. (**Table 1** and **Supplementary Table 1**).

**Table 1:** Characteristics of patients (N = 20).

Characteristics	N = 20 (*n*, %)
Gender (*n*, %)	
Male	10 (50)
Female	10 (50)
Primary disease (*n*, %)	
SAA	7 (35)
ALL	7 (35)
AML	4 (20)
MDS	2 (10)
Genes mutation/fusion genes	
Yes	12 (60)
No	8 (40)
Source of donors (*n*, %)	
Unrelated donors	10 (50)
Haploidentical donors	10 (50)
Conditioning regimens (*n*, %)	
TBI+FLU+BU+ Ara-c+ ALG	5 (25)
TBI + FLU + CTX+ ATG	5 (25)
FLU + BU + Ara-c + ALG	4 (20)
TBI + FLU + BU + Ara-C+VP16	3 (15)
FLU + CTX + ATG + Mel	1 (5)
TBI+FLU+CTX+ATG+Mel	1 (5)
FLU+BU+Mel	1 (5)
aGVHD prophylaxis (*n*, %)	
PTCY + ALG + CsA + MMF + ruxolitinib	13 (65)
PTCY + ALG + CsA + MMF + ruxolitinib + CBPSC	7 (35)
The median age (year)	12 (3–56)
The median number of reinfused MNCs (×10^8^/kg)	16.49 (5.67– 46.61)
The median number of reinfused CD34+ cells(× 10^6^/kg)	6.41 (2.13–15.7)
The median time for the neutrophil engraftment(d)	12 (10–15)
The median time for the platelet engraftment(d)	12 (7–15)

SAA, Severe aplastic anemia; AML Acute myeloid leukemia; ALL, Acute lymphocytic leukemia; MDS, Myelodysplastic syndrome; TBI, total-body irradiation; FLU, Fludarabine; BU, busulfan; Ara-c, Cytarabine; ALG, Antilymphocyte globulin; ATG, Antithymocyte globulin; VP16, Etoposide; Mel, Melphalan; GVHD, graft-versus-host disease; PTCY, posttransplant cyclophosphamide; CsA, cyclosporine A; MMF, mycophenolate mofetil; MNC, mononuclear cells; CBPSC, cord blood pluripotent stem cells.

### 3.2 Conditioning Regimens and GVHD Prophylaxis

In this study, the conditioning regimens included total-body irradiation (TBI) + fludarabine (FLU) + busulfan (BU) + cytarabine (Ara-c) + antilymphocyte globulin (ALG) in five cases, TBI + FLU + Cyclophosphamide (CTX) + antithymocyte globulin (ATG) in five cases, FLU + BU + Ara-c + ALG in four cases, TBI + FLU + BU + Ara-C+ etoposide (VP16) in three cases, FLU + CTX + ATG + melphalan (Mel) in one case, FLU+BU+Mel in one case and TBI+FLU+CTX+ATG+Mel in one case. For the aGVHD prophylaxis, 13 patients received CTX + ALG + cyclosporine A (CsA) + mycophenolate mofetil (MMF) + ruxolitinib, and seven patients received CTX + ALG + CsA + MMF + ruxolitinib + cord blood pluripotent stem cells (**Table 1** and **Supplementary Table 1**). Patients received 20 mg/kg CTX for the unrelated donor transplantation and 40 mg/kg for the haploidentical transplantation, at +3 d and +4 d. At -4 d to -1 d they received ALG, 12.0 mg/(kg · d) for the unrelated donor transplantation; at +8 d, 5 mg/(kg · d) for the haploidentical transplantation, and at +5 d, CsA and MMF were used. The initial dose of the CsA was 2 mg/kg.d for the adults and 2.5 mg/kg.d for the children. The dose was adjusted according to the concentrations of CsA. For the haploidentical transplant patients, the dose was reduced six months after the transplantation and stopped after 9–10 months. For the unrelated donor transplantation, the dose was reduced six months after the transplantation and stopped after 6–8 months. The plasma concentrations of CsA were assessed every three days and maintained at 150–250 ng/mL. The dosage of MMF was usually 500 mg twice a day, halved at four weeks after transplantation, and then stopped at six weeks. The concentrations of MMF were not assessed in all the patients. Ruxolitinib 5 mg/d and 0.07–0.1 mg/kg·d were administered to the adults and children, respectively, from the day of neutrophil engraftment to 100 days post-transplantation.

### 3.3 Hematopoietic Stem Cell Infusion and Engraftment

The median number of reinfused mononuclear cells (MNCs) and CD34+ cells were 16.49 (5.67– 46.61) ×10^8^/kg and 6.41 (2.13–15.7) × 10^6^/kg, respectively. The median time for the neutrophil and platelet engraftment were day 12 (10–15) and day 12 (7–15), respectively. (**Table 1** and **Supplementary Table 1**).

### 3.4 Symptoms of Central Nervous System and Laboratory Examination Results

**Figure 1:**
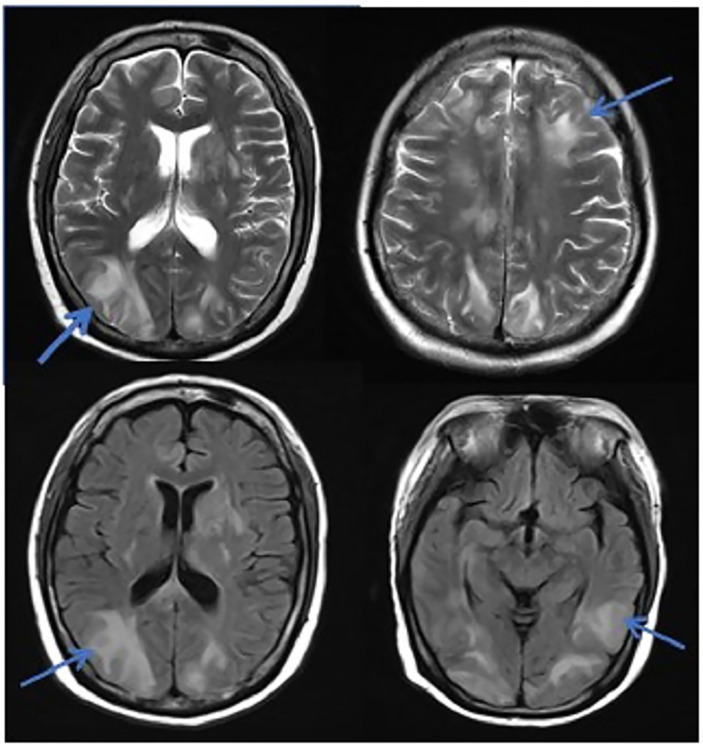
MRI in patients with PRES. The patient diagnosed with PRES showed a patchy low-intensity T1WI on the left occipital lobe, a high-intensity shadow on the T2WI and water pressure image, a high-intensity shadow in the DWI sequence part, unclear borders, and a shallow fission near the sulci.

**Figure 2:**
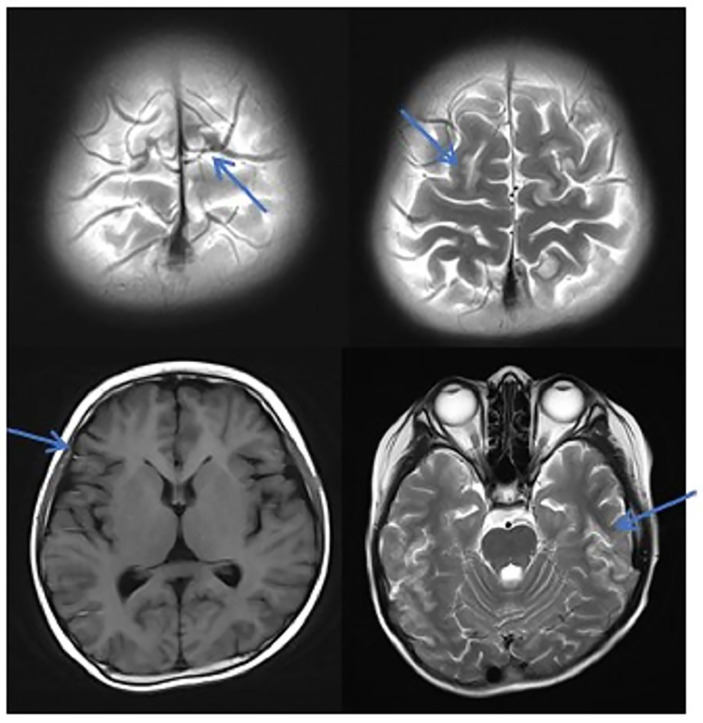
MRI in patients with TA-TMA. Patients with TA-TMA showed bilateral frontal, parietal, and occipital sulcus edges with a sheet-like low signal on the T1WI, a slightly high signal on the T2WI, a FLAIR high signal, unclear borders, a bilateral parietal dotted DWI high signal, and unclear borders.

**Figure 3:**
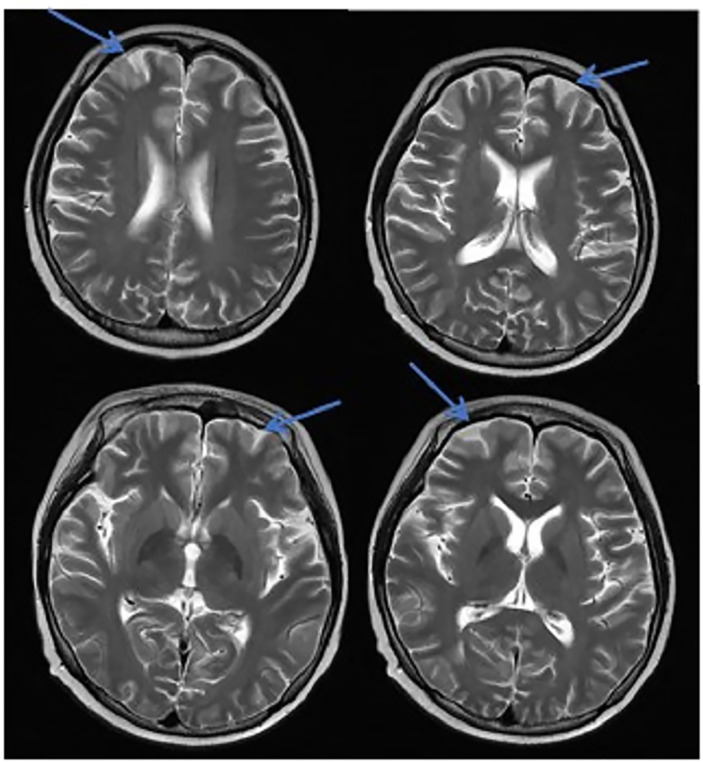
MRI in patients with CNS infection. The CNS infection showed flair hyperintensity in the cerebral cortex, but no clear display on the T2WI, T1WI, DWI, and no enhancement after enhancement.

**Figure 4:**
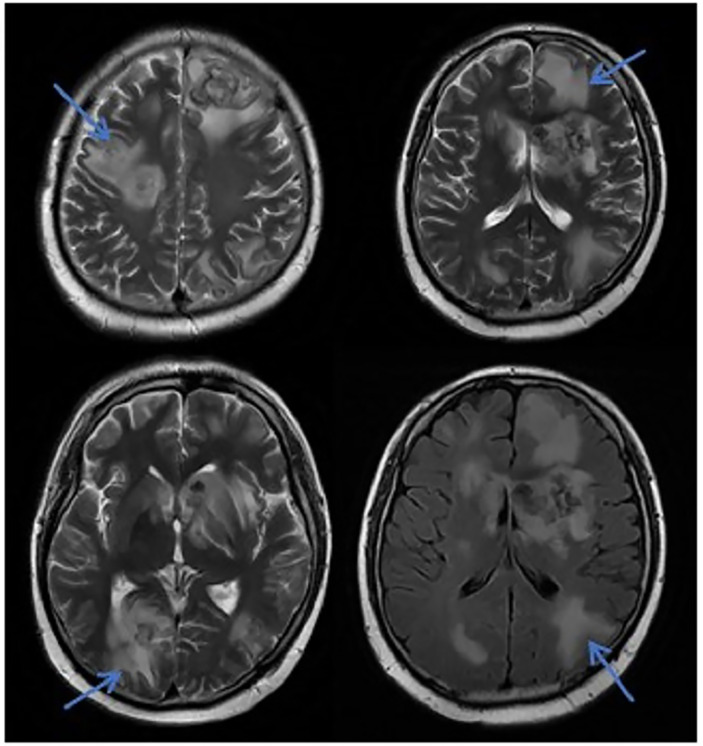
MRI in patients with CNS leukemia and infection. Primary central nervous system infiltration MRI indicated that the lesion showed mixed signals on the T1WI and nodules with mixed signals on the T2WI and T2WI flair. A DWI spot-like hyperintensity was observed in the lesion, and the enhancement.

The median time between the onset of the neurological symptoms and the transplantation was 64 (22–320) days, and 20 patients achieved hematopoietic reconstitution when they developed CNS symptoms. The symptoms of CNS included one case with a respiratory status change, three with fever, four with headache, 13 with mental status change, and 14 with convulsion. A total of 29 tests were performed with 21 of them being positive. Of the five negative patients, three cases were diagnosed as PRES (**Figure 1**), one as a TA-TMA (**Figure 2**), and one was considered as a transient seizure caused by hypertension. fifteen patients had infections, of which four were single infections and eleven mixed infections. Five cases of fungal infections, six cases of bacterial infections, and 13 cases of viral infections were detected (**Figure 3**). Among the 13 cases of viral infections, ten cases were CMV, three were BKPyV, and two were Torque teno virus(TTV), two were HHV-1, two were EBV and with one each of HpyV5 and HHV-6B. While all 13 of the patients had positive virus test results, six identical specimens tested negative for the virus using qPCR. Among the 15 patients with CNS infections, one had simultaneous central nervous system leukemia (**Figure 4**) and two had PRES. The results of the MRI, mNGS, bacterial cultures, acid-fast staining, and G experiments/GM experiments are shown in **Table 2**, **Supplementary Table 3** and **Figures 1** **–** **4**. All patients were tested by CSF smear, and no erythrocytes were detected. Peripheral blood contamination can be ruled out.

**Table 2:** Symptoms of CNS, imaging examination and prognosis (N = 20).

Case	Symptoms	Time from the onset of CNS symptom post-transplantation/d	mNGS			Brain MRI	Prognosis
			Virus	Bacteria	Fungus		
1	Fever, convulsion	107	+	+	+	Abnormal signal in frontal parietal lobe	Death
2	Convulsion	68	+	+	–	Abnormal signals in the cerebral cortex	Death
3	Convulsion, mental status change	51	–	–	–	Bilateral parietal abnormal signal	Survive
4	Breathing and mental status change	240	+	–	–	Subacute cerebral infarction	Death
5	Convulsion, headache	180	+	–	–	Abnormal signals in both cerebral hemispheres	Death
6	Convulsion	59	–	+	+	Bilateral frontal and parietal abnormal signal	Death
7	Mental status change	41	+	–	–	Bilateral parietal abnormal signal	Death
8	Convulsion	187	–	–	+	Abnormal signal in left basal ganglia	Death
9	Convulsion, mental status change	29	–	–	–	Abnormal signal of left frontal plate block	Survive
10	Fever, convulsion, mental status change	22	+	+	+	Abnormal signals in the center of the bilateral semioval and lateral ventricle	Death
						Formation of bilateral frontal subdural effusion	
11	Headache, Convulsion, mental status change	49	+	+	+	Left pituitary nodule	Survive
12	Mental status change	32	+	–	–	No data	Death
13	Fever, mental status change	215	+	–	–	No data	Death
14	Convulsion	97	–	–	–	Small vessel thrombosis	Death
15	Convulsion	181	–	–	–	Abnormal white matter signal in the right frontal lobe	Survive
16	Headache, mental status change	60	+	–	–	Extensive subcortical white matter lesions; subacute cerebral infarction	Death
17	Convulsion, mental status change	189	–	–	–	Bilateral frontal and parietal abnormal signal	Survive
18	Headache, mental status change	152	+	–	–	No data	Survive
19	Convulsion, mental status change	320	+	+	–	The sulci and the cistern were widened and deepened	Survive
20	Convulsion, mental status change	55	+	–	–	Multiple abnormal signals in the brain	Survive

+, Yes; -, No.

### 3.5 Other Transplant-Related Complications

Among the 20 patients, eight had aGVHD, of which five were grade II-IV aGVHD, two had cGVHD, 14 had pulmonary infections, nine had intestinal infections, seven had bacteremia, nine had hemorrhagic cystitis, seven had EBV infections, and 14 had CMV infections. (**Supplementary Table 2**). All those comorbidities were administered symptomatic and supportive treatment.

### 3.6 Treatment and Prognosis

According to the pathogen test results, drug sensitivities, and types of resistance, treatment was administered based on the Guidelines of the Infectious Diseases Working Party (AGIHO) of the German Society of Hematology and Medical Oncology (DGHO) (Schmidt-Hieber et al., 2016) and multi-center research experience (Lin and Liu, 2013; Gao et al., 2016). The neurological symptoms of ten patients improved after treatment, and the treatment effect was not obvious in 10 patients. As of July 2021, the median follow-up time after transplantation was 7.5 (2.1–24.5) months, and the median survival time of 15 patients with CNS infections was 30 (7–152) days. Twelve patients died, and the main cause of death was a nervous system infection in 10 patients, and severe pneumonia and organ failure in two patients. Eight patients survived. The survival rate of patients without CNS infection was 80% (4/5), and the survival rate of patients with CNS infection was only 26.7% (4/15).

## 4 Discussion

CNS infection was an important factor affecting patient survival. The overall mortality rates after development of encephalitis were for the most common viruses as follows: 67% for HHV-6, 83% for EBV, 80% for more than one detected virus (Schmidt-Hieber et al., 2011). Allo-HSCT patients are a high-risk group for CNS infections, with a total incidence of up to 15%, and most of them occurring within six months after transplantation (Pruitt et al., 2013). Studies have shown that 30% of patients with CNS complications after allo-HSCT had infections, of which 13% were bacterial, 10% fungal, and 7% viral (Colombo et al., 2017). The toxicities of pretreatment and GVHD preventive drugs can lead to a low immunity in patients after allo-HSCT and an increased risk of CNS infections. Once an infection occurs, it can lead to varying degrees of encephalitis, meningitis, and brain abscesses. Because the patients’ immune function is low, the early clinical symptoms are not obvious and are masked easily by other symptoms enabling the disease to progress rapidly. If the patient cannot be diagnosed and treated in time, the fatality rate is high (Schmidt-Hieber et al., 2016). In an autopsy study, more than 90% of the patients who died after allo-HSCT had neurological abnormalities (Weber et al., 2008). However, since allo-HSCT patients usually have multiple complications after transplantation, the symptoms of CNS lack specificity, making CNS infection an independent risk factor affecting the survival of allo-HSCT patients (Balaguer et al., 2017; Chaudhary et al., 2017). Hanajiri retrospectively analyzed the clinical data of 353 cases of CNS infections in allo-HSCT recipients. The median overall survival (OS) of patients after the CNS infections was 107 days, while the OS of patients with CNS infections was significantly lower than that of patients without CNS infections (Hanajiri et al., 2017). In our study, the survival rate of patients without CNS infection was 80%, but the survival rate of patients with CNS infection was only 26.7%. Therefore, the early, rapid, and precise diagnosis of CNS infections and pathogen types together with the implementation of timely, targeted treatment is essential for the improvement, survival, and prognosis of allo-HSCT patients.

Many methods such as G/GM experiments, ink staining, acid-fast staining, bacterial cultures, and quantitative PCR have been used for the diagnosis of CNS infections; However, they all have limitations. Although the conventional pathogen culture is the gold standard, it takes a long time and the rate of positivity is low. The G/GM experiments can only be used with low specificity and sensitivity for the preliminary approximate discrimination of fungi and the test results are usually affected by many factors. False positive and false negative results can occur, and the threshold for CSF detection is not clear. While acid-fast staining and ink staining can be used only for the detection of Mycobacterium tuberculosis and Cryptococcus, respectively, their sensitivities are low. Furthermore, although qPCR technology is widely used, its sensitivity is also low. There are difficulties in detecting viruses with a copy number of less than 10^3^ copies(U/ml), it is difficult to detect the early infections in time, and these methods can only detect specific pathogens, such as EBV, CMV, BKPyV, and adenovirus. At the same time, the number of specimens needed for detection is large, and it is difficult to obtain a sufficient amount of CSF in clinical practice. Therefore, traditional detection methods cannot detect pathogens early and accurately, and disease progression is often serious when discovered, which seriously affects the prognosis of CNS patients after allo-HSCT. The emergence of mNGS technology has solved these problems. There is no need to isolate and cultivate pathogens, and it can analyze multiple pathogens simultaneously. It has the advantages of a high detection sensitivity, short cycles, and a wide application range (Brown et al., 2018). In addition, the pathogen detection rate of mNGS is significantly better than that of traditional detection methods such as qPCR (Carpenter et al., 2019; Fei et al., 2020). mNGS’s unbiased approach broadens viral infection diagnosis, theoretically detecting “all” viral nucleotide sequences or viral infections present. Viral primary infections and reactivations are common complications after allo-HSCT and are associated with significant morbidity and mortality (Zanella et al., 2021). In our study, there were 13 viral infections, and most patients were mixed infections, the overall mortality of CNS infections after transplantation was 73.3%(11/15). The mortality was lower than previous research results (80%) (Schmidt-Hieber et al., 2011). Therefore, early accurate diagnosis and timely effective treatment are essential to improve the survival and prognosis of patients with CNS infection after transplantation.

In this study, due to the specificity of the patients’ primary disease treatment and the allo-HSCT, there were many reasons for the CNS symptoms of the patients. Approximately 61.1% of the CNS complications after allo-HSCT occurred within 100 days, such as PRES, GVHD, TA-TMA, and infections (Ke et al., 2019). The early clinical manifestations in these patients were not specific. Therefore, the diagnosis of the etiology of allo-HSCT with CNS symptoms requires the use of comprehensive auxiliary examination methods, especially in patients with CNS symptoms in the early stage. The MRI is the most commonly used detection method for CNS diseases. A variety of neurological diseases can be diagnosed initially through their MRI manifestations, while it needs to be combined with other detection methods for differential diagnosis. Research has shown that the MRI can be used as the diagnostic gold standard for PRES (Picchi et al., 2019).The MRI of the patient diagnosed with PRES in this study showed a patchy low-intensity T1WI on the left occipital lobe, a high-intensity shadow on the T2WI and water pressure image, a high-intensity shadow in the DWI sequence part, unclear borders, and a shallow fission near the sulci (**Figure 1**). The MRI in patient with TA-TMA showed bilateral frontal, parietal, and occipital sulcus edges with a sheet-like low signal on T1WI, a slightly high signal on T2WI, a FLAIR high signal, unclear borders, bilateral parietal dotted DWI high signal, and unclear borders (**Figure 2**). The primary central nervous system infiltration MRI showed a lesion with mixed signals on the T1WI and nodules with mixed signals on the T2WI and T2WI flair. A DWI spot-like hyperintensity was observed in the lesion, and an enhancement was observed after the enhancement (**Figure 4**). However, the MRI of the CNS infections showed flair hyperintensities in the cerebral cortexes, but no clear display on the T2WI, T1WI, DWI, and no enhancement after the enhancement (**Figure 3**). However, the results of MRI have limitations in the diagnosis of infections, and they are not specific, making it difficult to confirm the existence of infections and the types of infectious pathogens. Detection methods, such as the mNGS, CSF smear and bacterial culture, are needed to assist in the diagnoses.

There were no typical or specific changes in the MRI after CNS infections. In addition, due to the immunodeficiencies of the allo-HSCT patients, MRI imaging after CNS infections is even less specific. Even if the infection can be confirmed by an MRI, only the lesion of the infections can be observed, and the type of pathogen type causing the infections cannot be determined (Chaudhary et al., 2017). On the other hand, the mNGS method has a high positive rate for pathogen detection and strong reproducibility. The smallest unit of detection was one read. In particular, for the detection of virus infection and the sensitivity was significantly better than that of a traditional qPCR. In this study, among the 13 patients who tested positive for the virus, six identical specimens tested negative(<10^3^U/ml) for the virus using qPCR. For patients with fungal infections, the test results of the G/GM experiment were imprecise. The mNGS method can be used to perform untargeted and undifferentiated detections of specimens. It can not only target and identify specific microorganisms, but also detect different pathogens at the same time in one sequence, discover the sequences of known and unknown pathogens, and prioritize them according to the types of pathogens (Wilson et al., 2018). However, there are problems. Due to the high sensitivity, false-positive results may occur, and it is impossible to distinguish whether the detected pathogen is a pathogenic or parasitic bacterium. Therefore, it is not suitable for specimens that are easily contaminated by parasites, such as sputum and pharynx swabs. Therefore, attention must be paid to aseptic operations in the process of specimen collection, transportation, and storage. The CSF specimens used in this study were free of parasitic contamination. When obtaining cerebrospinal fluid during a lumbar puncture, it is necessary to pay attention to the disinfection of the local skin; CSF smear was tested to avoid the cerebrospinal fluid being mixed with blood, leading to false positive results. Simultaneous mNGS testing of the CSF and peripheral blood can be used to overcome this. However, this may increase the financial burden on patients. Overall, the application of the mNGS method has greatly improved the detection rate of patients with CNS infections after transplantation (Boers et al., 2019). An early and efficient diagnosis not only enables patients to receive timely and targeted treatment, to buy time for patients treatment, but is also essential to improve the prognosis of patients and to increase the survival rate of patients with CNS infections after transplantation (Schmidt-Hieber et al., 2020).

The mNGS method detects pathogens quickly, has a high sensitivity, and a wide detection range for microorganisms. It can assist in the early diagnosis of CNS infections after allo-HSCT and provide a basis for the timely selection of effective anti-infective drugs. However, clinicians still need to be cautious when diagnosing CNS infections. They need to use a combination of clinical symptoms, imaging examinations, bacteriological cultures, mNGS techniques, and other auxiliary testing methods. They also need to adhere strictly to the principles of sterility during specimen collection, transportation, and testing. Simultaneously, to obtain more accurate test results, blood samples and CSF smear should be tested to exclude contamination and false positive results. In addition, large samples, multi-center, prospective studies are needed to confirm the application standards and timing of the use of the mNGS method in patients with CNS symptoms after hematopoietic stem cell transplantation.

## Data Availability Statement

All the data generated or analyzed during this study are included in this published article. Genome sequences had been uploaded into NCBI (BioProject ID: PRJNA756408).

## Ethics Statement

The studies involving human participants were reviewed and approved by Ethics Committee of the Affiliated Cancer Hospital of Zhengzhou University. Written informed consent to participate in this study was provided by the participants’ legal guardian/next of kin. Written informed consent was obtained from the individual(s), and minor(s)’ legal guardian/next of kin, for the publication of any potentially identifiable images or data included in this article.

## Author Contributions

BZ, JZ, and YS designed the study. RG, ZL, YZu, JW, and HZ implemented this research. BZ, YZh, and ZJ collected medical records. BZ, JZ, and YS drafted the manuscript. All the authors participated in the revision of the manuscript. All authors contributed to the article and approved the submitted version.

## Funding

This study was supported, in part, by the Affiliated Cancer Hospital of Zhengzhou University, Zhengzhou, China.

## Conflict of Interest

The authors declare that the research was conducted in the absence of any commercial or financial relationships that could be construed as a potential conflict of interest.

## Publisher’s Note

All claims expressed in this article are solely those of the authors and do not necessarily represent those of their affiliated organizations, or those of the publisher, the editors and the reviewers. Any product that may be evaluated in this article, or claim that may be made by its manufacturer, is not guaranteed or endorsed by the publisher.

## Abbreviations

CNS, central nervous system; allo-HSCT, allogeneic hematopoietic stem cell transplantation; TA-TMA, transplant-related thrombotic microangiopathies; GVHD, graft versus host disease; OS, overall survival; CMV, cytomegalovirus; EBV, Epstein-Barr virus; HHV, Human alphaherpesvirus; TTV, Torque teno virus; HpyV5, Human polyomavirus 5; SAA, severe aplastic anemia; ALL, acute lymphoblastic leukemia; AML, acute myeloid leukemia; CML, chronic myeloid leukemia; MDS, myelodysplastic syndrome; TBI, total-body irradiation; FLU, fludarabine; BU, busulfan; Ara-c, cytarabine; ALG, antilymphocyte globulin; ATG, antithymocyte globulin; VP16, etoposide; Mel melphalan; CsA, cyclosporine A; MMF, mycophenolate mofetil; MNC, mononuclear cells; PRES, posterior reversible encephalopathy syndrome.

## References

[B1] Balaguer R. A. Bataller L. Lorenzo I. Jarque I. Salavert M. Gonzalez E.. (2017). Infections of the Central Nervous System After Unrelated Donor Umbilical Cord Blood Transplantation or Human Leukocyte Antigen-Matched Sibling Transplantation. Biol. Blood Marrow Transplant. 23 (1), 134–139. doi: 10.1016/j.bbmt.2016.10.005 27794456

[B2] Balaguer-Rosello A. Bataller L. Pinana J. L. Montoro J. Lorenzo I. Villalba A.. (2019). Noninfectious Neurologic Complications After Allogeneic Hematopoietic Stem Cell Transplantation. Biol. Blood Marrow Transplant. 25 (9), 1818–1824. doi: 10.1016/j.bbmt.2019.05.024 31132454

[B3] Boers S. A. Jansen R. Hays J. P. (2019). Understanding and Overcoming the Pitfalls and Biases of Next-Generation Sequencing (Ngs) Methods for Use in the Routine Clinical Microbiological Diagnostic Laboratory. Eur. J. Clin. Microbiol. Infect. Dis. 38 (6), 1059–1070. doi: 10.1007/s10096-019-03520-3 30834996 PMC6520317

[B4] Brown J. R. Bharucha T. Breuer J. (2018). Encephalitis Diagnosis Using Metagenomics: Application of Next Generation Sequencing for Undiagnosed Cases. J. Infect. 76 (3), 225–240. doi: 10.1016/j.jinf.2017.12.014 29305150 PMC7112567

[B5] Carpenter M. L. Tan S. K. Watson T. Bacher R. Nagesh V. Watts A.. (2019). Metagenomic Next-Generation Sequencing for Identification and Quantitation of Transplant-Related DNA Viruses. J. Clin. Microbiol. 57 (12). doi: 10.1128/JCM.01113-19 PMC6879295 31554674

[B6] Chaudhary R. K. Dhakal P. Aryal A. Bhatt V. R. (2017). Central Nervous System Complications After Allogeneic Hematopoietic Stem Cell Transplantation. Future Oncol. 13 (25), 2297–2312. doi: 10.2217/fon-2017-0274 28984145

[B7] Chen S. Zhou Y. Chen Y. Gu J. (2018). Fastp: An Ultra-Fast All-in-One Fastq Preprocessor. Bioinformatics 34 (17), i884–i890. doi: 10.1093/bioinformatics/bty560 30423086 PMC6129281

[B8] Colombo A. A. Marchioni E. Diamanti L. Di Matteo A. M. Baldanti F. Furione M.. (2017). Neurological Complications Involving the Central Nervous System After Allogeneic Hematopoietic Stem Cell Transplantation During a Period of Evolution in Transplant Modalities: A Cohort Analysis. Transplantation 101 (3), 616–623. doi: 10.1097/TP.0000000000001257 27222935

[B9] Das J. Gill A. Lo C. Chan-Lam N. Price S. Wharton S. B.. (2020). A Case of Multiple Sclerosis-Like Relapsing Remitting Encephalomyelitis Following Allogeneic Hematopoietic Stem Cell Transplantation and a Review of the Published Literature. Front. Immunol. 11:668. doi: 10.3389/fimmu.2020.00668 32431694 PMC7214636

[B10] Dobin A. Davis C. A. Schlesinger F. Drenkow J. Zaleski C. Jha S.. (2013). Star: Ultrafast Universal RNA-Seq Aligner. Bioinformatics 29 (1), 15–21. doi: 10.1093/bioinformatics/bts635 23104886 PMC3530905

[B11] Dowling M. R. Li S. Dey B. R. McAfee S. L. Hock H. R. Spitzer T. R.. (2018). Neurologic Complications After Allogeneic Hematopoietic Stem Cell Transplantation: Risk Factors and Impact. Bone Marrow Transplant. 53 (2), 199–206. doi: 10.1038/bmt.2017.239 29131150

[B12] Fei X. Li C. Zhang Y. Zhang H. Liu X. Ji X.. (2020). Next-Generation Sequencing of Cerebrospinal Fluid for the Diagnosis of Neurocysticercosis. Clin. Neurol. Neurosurg. 193:105752. doi: 10.1016/j.clineuro.2020.105752 32220712

[B13] Gao L. Sun Y. Meng F. Han M. Huang H. Wu D.. (2016). Antifungal Prophylaxis of Patients Undergoing Allogenetic Hematopoietic Stem Cell Transplantation in China: A Multicenter Prospective Observational Study. J. Hematol. Oncol. 9 (1), 97. doi: 10.1186/s13045-016-0305-y 27663309 PMC5035465

[B14] Hanajiri R. Kobayashi T. Yoshioka K. Watanabe D. Watakabe K. Murata Y.. (2017). Central Nervous System Infection Following Allogeneic Hematopoietic Stem Cell Transplantation. Hematol. Oncol. Stem Cell Ther. 10 (1), 22–28. doi: 10.1016/j.hemonc.2016.08.008 27664550

[B15] Haston J. C. Rostad C. A. Jerris R. C. Milla S. S. McCracken C. Pratt C.. (2020). Prospective Cohort Study of Next-Generation Sequencing as a Diagnostic Modality for Unexplained Encephalitis in Children. J. Pediatr. Infect. Dis. Soc 9 (3), 326–333. doi: 10.1093/jpids/piz032 PMC7457329 31107955

[B16] Ke P. Bao X. Qiu H. Zhuang J. Hu X. Wu X.. (2019). Central Nervous System Complications Caused by 3-4 Grade Agvhd in Adult Patients Occurred in Hla-Mismatched Recipients Majorly After Allogeneic Hematopoietic Stem Cell Transplantation. Bone Marrow Transplant. 54 (7), 1155–1157. doi: 10.1038/s41409-019-0443-2 30664722

[B17] Lin R. Liu Q. (2013). Diagnosis and Treatment of Viral Diseases in Recipients of Allogeneic Hematopoietic Stem Cell Transplantation. J. Hematol. Oncol. 6:94. doi: 10.1186/1756-8722-6-94 24341630 PMC3878524

[B18] Maffini E. Festuccia M. Brunello L. Boccadoro M. Giaccone L. Bruno B. (2017). Neurologic Complications After Allogeneic Hematopoietic Stem Cell Transplantation. Biol. Blood Marrow Transplant. 23 (3), 388–397. doi: 10.1016/j.bbmt.2016.12.632 28039081

[B19] Picchi E. Di Giuliano F. Marziali S. Minosse S. Ferrazzoli V. Da R. V.. (2019). Radiological Findings of Posterior Reversible Encephalopathy Syndrome in Transplanted Children Previous Affected by Hemoglobinopathy: A Neuroimaging Retrospective Analysis. Eur. J. Radiol. Open 6, 144–151. doi: 10.1016/j.ejro.2019.03.001 31016209 PMC6468159

[B20] Pruitt A. A. Graus F. Rosenfeld M. R. (2013). Neurological Complications of Transplantation: Part I: Hematopoietic Cell Transplantation. Neurohospitalist 3 (1), 24–38. doi: 10.1177/1941874412455338 23983885 PMC3726122

[B21] Schmidt-Hieber M. Engelhard D. Ullmann A. Ljungman P. Maertens J. Martino R.. (2020). Central Nervous System Disorders After Hematopoietic Stem Cell Transplantation: A Prospective Study of the Infectious Diseases Working Party of Ebmt. J. Neurol. 267 (2), 430–439. doi: 10.1007/s00415-019-09578-5 31664549

[B22] Schmidt-Hieber M. Schwender J. Heinz W. J. Zabelina T. Kuhl J. S. Mousset S.. (2011). Viral Encephalitis After Allogeneic Stem Cell Transplantation: A Rare Complication With Distinct Characteristics of Different Causative Agents. Haematologica 96 (1), 142–149. doi: 10.3324/haematol.2010.029876 20851868 PMC3012778

[B23] Schmidt-Hieber M. Silling G. Schalk E. Heinz W. Panse J. Penack O.. (2016). Cns Infections in Patients With Hematological Disorders (Including Allogeneic Stem-Cell Transplantation)-Guidelines of the Infectious Diseases Working Party (Agiho) of the German Society of Hematology and Medical Oncology (Dgho). Ann. Oncol. 27 (7), 1207–1225. doi: 10.1093/annonc/mdw155 27052648 PMC4922317

[B24] Schmieder R. Edwards R. (2011). Quality Control and Preprocessing of Metagenomic Datasets. Bioinformatics 27 (6), 863–864. doi: 10.1093/bioinformatics/btr026 21278185 PMC3051327

[B25] Weber C. Schaper J. Tibussek D. Adams O. Mackenzie C. R. Dilloo D.. (2008). Diagnostic and Therapeutic Implications of Neurological Complications Following Paediatric Haematopoietic Stem Cell Transplantation. Bone Marrow Transplant. 41 (3), 253–259. doi: 10.1038/sj.bmt.1705905 17982498

[B26] Wilson M. R. Naccache S. N. Samayoa E. Biagtan M. Bashir H. Yu G.. (2014). Actionable Diagnosis of Neuroleptospirosis by Next-Generation Sequencing. N Engl. J. Med. 370 (25), 2408–2417. doi: 10.1056/NEJMoa1401268 24896819 PMC4134948

[B27] Wilson M. R. O’Donovan B. D. Gelfand J. M. Sample H. A. Chow F. C. Betjemann J. P.. (2018). Chronic Meningitis Investigated via Metagenomic Next-Generation Sequencing. JAMA Neurol. 75 (8), 947–955. doi: 10.1001/jamaneurol.2018.0463 29710329 PMC5933460

[B28] Wood D. E. Lu J. Langmead B. (2019). Improved Metagenomic Analysis With Kraken 2. Genome Biol. 20 (1), 257. doi: 10.1186/s13059-019-1891-0 31779668 PMC6883579

[B29] Xing X. W. Zhang J. T. Ma Y. B. He M. W. Yao G. E. Wang W.. (2020). Metagenomic Next-Generation Sequencing for Diagnosis of Infectious Encephalitis and Meningitis: A Large, Prospective Case Series of 213 Patients. Front. Cell Infect. Microbiol. 10:88. doi: 10.3389/fcimb.2020.00088 32211343 PMC7066979

[B30] Zanella M. C. Cordey S. Laubscher F. Docquier M. Vieille G. Van Delden C.. (2021). Unmasking Viral Sequences by Metagenomic Next-Generation Sequencing in Adult Human Blood Samples During Steroid-Refractory/Dependent Graft-*Versus*-Host Disease. Microbiome 9 (1), 28. doi: 10.1186/s40168-020-00953-3 33487167 PMC7831233

[B31] Zhang Y. Cui P. Zhang H. C. Wu H. L. Ye M. Z. Zhu Y. M.. (2020). Clinical Application and Evaluation of Metagenomic Next-Generation Sequencing in Suspected Adult Central Nervous System Infection. J. Transl. Med. 18 (1), 199. doi: 10.1186/s12967-020-02360-6 32404108 PMC7222471

[B32] Zhang X. X. Guo L. Y. Liu L. L. Shen A. Feng W. Y. Huang W. H.. (2019). The Diagnostic Value of Metagenomic Next-Generation Sequencing for Identifying Streptococcus Pneumoniae in Paediatric Bacterial Meningitis. BMC Infect. Dis. 19 (1), 495. doi: 10.1186/s12879-019-4132-y 31164085 PMC6549306

