# Supplementary material for: Metagenomic Next Generation Sequencing in the Detection of Pathogens in Cerebrospinal Fluid of Patients After Alternative Donor Transplantation: A Feasibility Analysis
Source: Front Cell Infect Microbiol. 2021 Sep 14;11:720132. doi: 10.3389/fcimb.2021.720132 (PMC8476959; doi:10.3389/fcimb.2021.720132)
Supplement: Supplementary file 1 [file Table_1.docx]

Table1 characteristics of patients (N=20)

| Case | Gender | Age（year） | Primary disease | Donor source | HLA matching | Gene mutation or fusion gene | Abnormal chromosome | Conditioning regimen | GVHD prophylaxis | MNC（10^8^/kg） | CD34+（10^6^/kg） | Time for engraftment of neutrophils (d) | Time for engraftment of platelets (d) |
| --- | --- | --- | --- | --- | --- | --- | --- | --- | --- | --- | --- | --- | --- |
| 1 | Female | 8 | SAA | HPD | 8/10 | - | - | TBI+FLU+CTX+ATG | PTCY+CSA+MMF+ALG+Ruxolitinib | 26.52 | 8.7 | 14 | 12 |
| 2 | Female | 13 | SAA | HPD | 5/10 | TERT/EXON7 | - | TBI+FLU+CTX+ATG | PTCY+CSA+MMF+ALG+Ruxolitinib | 20.59 | 4.49 | 12 | 15 |
| 3 | Male | 13 | SAA | HPD | 8/10 | - | - | TBI+FLU+CTX+ATG | PTCY+CSA+MMF+ALG+Ruxolitinib | 16.82 | 5.44 | 11 | 7 |
| 4 | Male | 30 | AML | HPD | 8/10 | CEBPA/TET2 | - | TBI+FLU+BU+Ara-c+ALG | PTCY+CSA+MMF+ALG+Ruxolitinib | 9.81 | 6.13 | 12 | 12 |
| 5 | Male | 18 | ALL | URD | 10/10 | FLT3/ITD | - | TBI+FLU+BU+Ara-c+ALG | PTCY+CSA+MMF+ALG+Ruxolitinib | 15.22 | 2.13 | 13 | 14 |
| 6 | Female | 30 | ALL | URD | 9/10 | - | - | TBI+FLU+BU+Ara-c+ALG | PTCY+CSA+MMF+ALG+Ruxolitinib | 14.07 | 5.63 | 11 | 11 |
| 7 | Male | 11 | ALL | HPD | 5/10 | NRAS/IKZF1/  ABCB1 | 52,XY,dup（1）（q10，q42）*2；+2，+4，+6，+7，+13，+16[cp10] | TBI+FLU+BU+Ara-C+VP16 | PTCY+CSA+MMF+ALG+Ruxolitinib | 46.61 | 5.68 | 12 | 13 |
| 8 | Female | 56 | ALL | URD | 9/10 | E2A/PBX1 | - | FLU+BU+Ara-c+ALG | PTCY+CSA+MMF+ALG+Ruxolitinib | 16.17 | 11.48 | 11 | 9 |
| 9 | Male | 8 | AML | HPD | 6/10 | WT1/MLL,  AF6/NRAS | 46,XY,T(6;11)(q25;q23）[2]/48,idem,+8,+21[18] | TBI+FLU+BU+Ara-C+VP16 | PTCY+CSA+MMF+ALG+Ruxolitinib | 18.89 | 13.03 | 13 | 11 |
| 10 | Female | 3 | AML | HPD | 5/10 | MLL-AF10/FLT3-TKD/TET2/NRS | 46,XX,t(10；11)(p11.2，q13)[10] | TBI+FLU+BU+Ara-C+VP16 | PTCY+CSA+MMF+ALG+Ruxolitinib+CBSC | 13.08 | 10.34 | 13 | 13 |
| 11 | Female | 8 | ALL | URD | 10/10 | BCR/ABL | 47,XX,+1(q10),t(9,22)(q34，q11)[2] | TBI+FLU+BU+Ara-c+ALG | PTCY+CSA+MMF+ALG+Ruxolitinib+CBSC | 12.2 | 4.39 | 10 | 10 |
| 12 | Male | 36 | ALL | URD | 8/10 | - | - | TBI+FLU+BU+Ara-c+ALG | PTCY+CSA+MMF+ALG+Ruxolitinib+CBSC | 5.67 | 2.89 | 12 | 12 |
| 13 | Male | 11 | SAA | HPD | 5/10 | - | - | TBI+FLU+CTX+ATG | PTCY+CSA+MMF+ALG+Ruxolitinib+CBSC | 24.98 | 6.93 | 12 | 11 |
| 14 | Male | 6 | ALL | URD | 10/10 | IKZF1 | 60,XY,+X,+1,+4,+6,+8+10+11,+12,+18,+19,+20,+21,+22,+22[1]/46XY[8] | FLU+BU+Ara-c+ALG | PTCY+CSA+MMF+ALG+Ruxolitinib | 29.3 | 15.7 | 11 | 12 |
| 15 | Female | 8 | MDS | URD | 9/10 | SETBP1 | 45,XX,-7/-7q,inc[5] | FLU+BU+Ara-c+ALG | PTCY+CSA+MMF+ALG+Ruxolitinib | 20.06 | 8.02 | 14 | 14 |
| 16 | Female | 42 | SAA | URD | 9/10 | - | - | FLU+CTX+ATG+Mel | PTCY+CSA+MMF+ALG+Ruxolitinib+CBSC | 7.99 | 3.75 | 10 | 11 |
| 17 | Female | 9 | SAA | HPD | 5/10 | - | - | TBI+FLU+CTX+ATG | PT-Cy+CSA+MMF+ALG+ Ruxolitinib | 23.37 | 5.27 | 11 | 12 |
| 18 | Female | 33 | AML | URD | 9/10 | BCR/ABL210 | t(9;22)(q34;q11) | FLU+BU+Ara-c+ALG | PTCY+CSA+MMF+ALG+Ruxolitinib+CBSC | 20.25 | 6.68 | 12 | 13 |
| 19 | Male | 7 | SAA | HPD | 5/10 | SETBP-1/ETV6 | 45,XY,-7[10] | Flu+BU+Mel | PTCY+CSA+MMF+ALG+Ruxolitinib+CBSC | 9.42 | 7.6 | 15 | 11 |
| 20 | Male | 31 | MDS | URD | 9/10 | - | - | TBI+FLU+CTX+Mel+ATG | PTCY+CSA+MMF+ALG+Ruxolitinib | 7.78 | 9.26 | 11 | 15 |

SAA, Severe aplastic anemia; AML Acute myeloid leukemia; ALL, Acute lymphocytic leukemia; MDS, Myelodysplastic syndrome; URD, Unrelated donors; HPD, haploidentical donors; TBI, total-body irradiation; FLU, Fludarabine; BU, busulfan; Ara-c, Cytarabine; ALG, Antilymphocyte globulin; ATG, Antithymocyte globulin; VP16, Etoposide; Mel, Melphalan; GVHD, graft-versus-host disease; PTCY, posttransplant cyclophosphamide; CsA, cyclosporine A; MMF, mycophenolate mofetil; MNC, mononuclear cells;

Table2 Patients with other transplant-related complications (N=20)

| Case | aGVHD | Grade II-IV aGVHD | cGVHD | pulmonary infection | intestinal infection | bacteremia | hemorrhagic cystitis | EBV infection | CMV infection |
| --- | --- | --- | --- | --- | --- | --- | --- | --- | --- |
| 1 | + | - | - | + | - | + | + | + | + |
| 2 | + | II | - | - | - | + | - | + | + |
| 3 | - | - | - | - | - | - | - | + | + |
| 4 | - | - | + | + | + | - | + | + | + |
| 5 | + | - | - | + | + | - | + | - | + |
| 6 | - | - | - | + | - | - | + | - | + |
| 7 | - | - | - | - | - | - | - | - | - |
| 8 | - | - | - | + | + | + | + | - | + |
| 9 | - | - | - | + | - | - | - | - | - |
| 10 | + | III | + | + | + | + | - | - | + |
| 11 | - | - | - | + | + | + | - | - | - |
| 12 | - | - | - | + | + | - | + | + | + |
| 13 | + | II | - | + | - | - | + | + | + |
| 14 | - | - | - | + | - | + | - | - | + |
| 15 | - | - | - | - | - | - | - | - | + |
| 16 | - | - | - | + | + | - | - | + | + |
| 17 | - | - | - | + | + | - | + | - | - |
| 18 | + | - | - | - | - | + | + | - | + |
| 19 | + | II | - | + | + | - | - | - | - |
| 20 | + | II | - | - | - | - | - | - | - |

GVHD, graft-versus-host disease; EBV, Epstein-Barr virus ; CMV, cytomegalovirus

Table3 Symptoms of CNS, results of laboratory, imaging examination and prognosis (N=20)

| Number | case | symptoms | Time from the onset of CNS symptom post-transplantation/d | mNGS | | | | | | qPCR | | G experiments | GM experiments | ink staining | acid-fast staining | bacterial cultures | Brain MRI | Prognosis |
| --- | --- | --- | --- | --- | --- | --- | --- | --- | --- | --- | --- | --- | --- | --- | --- | --- | --- | --- |
|  |  |  |  | Virus | reads | Bacteria | reads | Fungus | reads | virus | U/ml | Fungus（pg/ml) | Fungus(ug/L) | Cryptococcus neoformans | Mycobacteri | G+/G- bacteria |  |  |
| 19PM0369 | 1.1 | Fever, convulsion | 107 | BKpyV | 5 |  |  |  |  | BKpyV | ＜1×10^3^ |  |  | N | N | N | Abnormal signal in frontal parietal lobe | Death |
| 19PM0808 | 1.2 |  |  | N |  | N |  | Fusarium verticillioides | 4 |  |  | 31 | 0.27 | N | N | N |  |  |
| 19PM0901 | 1.3 |  |  | N |  | Klebsiella pneumoniae | 682 | N |  |  |  |  |  | N | N | N |  |  |
| 19PM0385 | 2 | Convulsion | 68 | EBV(HHV-4) | 6 | Staphylococ-cus haemolyticus | 41 | N |  | EBV(HHV-4) | ＜1×10^3^ |  |  | N | N | N | Abnormal signals in the cerebral cortex | Death |
|  |  |  |  | N |  | Staphylococcus hominis | 28 |  |  |  |  |  |  | N | N | N |  |  |
| 19PM0430 | 3 | Convulsion, mental status change | 51 | N |  | N |  | N |  |  |  |  |  | N | N | N | Bilateral parietal abnormal signal | Survive |
| 19PM0484 | 4 | Breathing and mental status change | 240 | BKpyV | 440 | N |  | N |  | BKpyV | 8×10^3^ |  |  | N | N | N | Subacute cerebral infarction | Death |
|  |  |  |  | CMV(HHV-5) | 335 | N |  | N |  | CMV（HHV-5) | 6.5×10^3^ |  |  | N | N | N |  |  |
| 19PM0581 | 5 | Convulsion, headache | 180 | CMV（HHV-5) | 3 | N |  | N |  | CMV（HHV-5) | ＜1×10^3^ | - | - | N | N | N | Abnormal signals in both cerebral hemispheres | Death |
|  |  |  |  | BKpyV | 9 |  |  |  |  | BKpyV | ＜1×10^3^ |  |  |  |  |  |  |  |
| 20PM00284 | 6 | Convulsion | 59 | N |  | stenotrophomonas maltophilia | 4166 | Aspergillus sp. | 907 |  |  | 99 | 1.13 | N | N | N | Bilateral frontal and parietal abnormal signal | Death |
| 20PM00350 | 7 | Mental status change | 41 | CMV（HHV-5) | 26568 | N |  | N |  | CMV（HHV-5) | 2.23×10^4^ | - | - | N | N | N | Bilateral parietal abnormal signal | Death |
| 20PM00365 | 8 | Convulsion | 187 | N |  | N |  | Aspergillus niger | 15 | - |  | 41 | 0.32 | N | N | N | Abnormal signal in left basal ganglia | Death |
| 20PM00366 | 9 | Convulsion, mental status change | 29 | N |  | N |  | N |  | - |  | - | - | N | N | N | Abnormal signal of left frontal plate block | Survive |
| 20PM00446 | 10.1 | Fever, convulsion, mental status change | 22 | CMV（HHV-5) | 23 | Acinetobacte rbaumannii | 3763 | Fusarium oxysporum | 26 | CMV（HHV-5) | 2.46×10^3^ | 56 | 0.49 | N | N | N | Abnormal signals in the center of the bilateral semioval and lateral ventricle, formation of bilateral frontal subdural effusion | Death |
|  |  |  |  | N |  | N |  | Penicillium chrysogenum | 26 |  |  | 59 | 0.46 | N | N | N |  |  |
| 20PM00500 | 10.2 |  |  | CMV（HHV-5) | 10 | N |  | Penicillium chrysogenum | 45 | CMV（HHV-5) | 1.78×10^3^ | 85 | 0.76 | N | N | N |  |  |
| 20PM00648 | 10.3 |  |  | N |  | N |  | N |  |  |  |  |  | N | N | N |  |  |
| 20PM00826 | 10.4 |  |  | N |  | N |  | N |  |  |  |  |  | N | N | N |  |  |
| 20PM00542 | 10.5 |  |  | CMV（HHV-5) | 14 | N |  | Penicillium chrysogenum | 43 | CMV（HHV-5) | 1.97×10^3^ | 88 | 0.74 | N | N | N |  |  |
| 20PM100807 | 11.1 | Headache, Convulsion, mental status change | 49 | N |  | Leuconostoc pseudomesenteroides | 19874 | N |  |  |  |  |  | N | N | N | Left pituitary nodule | Survive |
| 20PM100934 | 11.2 |  |  | N |  | N |  | Fusarium oxysporum | 47 |  |  | 89 | 0.78 | N | N | N |  |  |
| MBX24507 | 11.3 |  |  | CMV（HHV-5) | 3 | N |  | Rhizopus delemar | 1 | CMV（HHV-5) | ＜1×10^3^ | 23 | 0.22 | N | N | N |  |  |
| MBX24352 | 11.4 |  |  | N |  | N |  | N |  |  |  |  |  | N | N | N |  |  |
| 20PM100817 | 12 | Mental status change | 32 | HHV-1 | 313 | N |  | N |  | HHV-1 | 5.91×10^3^ |  | - | N | N | N | No data | Death |
|  |  |  |  | CMV（HHV-5) | 4 |  |  |  |  | CMV（HHV-5) | ＜1×10^3^ |  |  |  |  |  |  |  |
| 20PM00521 | 13 | Fever, mental status change | 215 | EBV(HHV-4) | 59 | N |  | N |  | EBV(HHV-4) | 3.87×10^3^ | - | - | N | N | N | No data | Death |
| 21PM100063 | 14 | Convulsion | 97 | N |  | N |  | N |  | - |  | - | - | N | N | N | Small vessel thrombosis | Death |
| 21PM101217 | 15 | Convulsion | 181 | N |  | N |  | N |  | - |  | - | - | N | N | N | Abnormal white matter signal in the right frontal lobe | Survive |
| MBX11045 | 16 | Headache, mental status change | 60 | TTV | 6 | N |  | N |  | TTV | ＜1×10^3^ | - | - | N | N | N | Extensive subcortical white matter lesions; subacute cerebral infarction | Death |
|  |  |  |  | CMV(HHV-5) | 2 |  |  |  |  | CMV（HHV-5) | ＜1×10^3^ |  |  |  |  |  |  |  |
| NGS116 | 17 | Convulsion, mental status change | 189 | N |  | N |  | N |  | - |  | - | - | N | N | N | Bilateral frontal and parietal abnormal signal | Survive |
| NGS144 | 18 | Headache, mental status change | 152 | CMV（HHV-5) | 529798 | N |  | N |  | CMV（HHV-5) | 4.86×10^4^ | - | - | N | N | N | No data | Survive |
|  |  |  |  | HHV-6B | 3 |  |  |  |  | HHV-6B | ＜1×10^3^ |  |  |  |  |  |  |  |
| NGS223 | 19 | Convulsion, mental status change | 320 | HHV-1 | 5 | Enterococcus faecium | 77 | N |  | HHV-1 | ＜1×10^3^ | - | - | N | N | N | The sulci and the cistern were widened and deepened | Survive |
|  |  |  |  | HpyV5 | 1 |  |  |  |  | HpyV5 | ＜1×10^3^ |  |  |  |  |  |  |  |
| NGS237 | 20 | Convulsion, mental status change | 55 | TTV | 165 | N |  | N |  | TTV | 3.54×10^3^ | - | - | N | N | N | Multiple abnormal signals in the brain | Survive |

CNS, Central nervous system; mNGS, metagenomic next generation sequencing; EBV, Epstein-Barr virus ; CMV, cytomegalovirus; HHV, Human alphaherpesvirus; TTV, Torque teno virus; HpyV5, Human polyomavirus 5; N, No;
